# Supplementary material for: Quantum Computing Based Design of Multivariate Porous Materials
Source: ACS Cent Sci. 2025 Aug 22;11(10):1921–32. doi: 10.1021/acscentsci.5c00918 (PMC12550618; doi:10.1021/acscentsci.5c00918)
Supplement: Supplementary file 1 [file oc5c00918_si_001.pdf]

Supporting Information for:

Quantum Computing Based Design of Multivariate Porous Materials

Shinyoung Kang, Younghun Kim, Jihan Kim*

Department of Chemical and Biomolecular Engineering, Korea Advanced Institute of Science and Technology, 291 Daehak-ro, Yuseong-gu, Daejeon 34141, Republic of Korea

Table of Contents

Table S1 Glossary of Acronyms Page 3

Note S1 Comparative analysis of balance cost as a function of α Page 4

Figure S1 Balance cost trends at varying values of α in the Hamiltonian Page 6

Figure S2 Illustration of the characteristic lengths of ditopic and tritopic linkers Page 7

Figure S3 $G(i,j,w_{i,j})$ of candidate experimental structures Page 8

Note S2 Computational method for Sampling VQE simulation Page 9

Table S2 Characteristic lengths of candidate linkers Page 11

Table S3 Summary of parameters for $G(i,j,w_{i,j})$ Page 11

Figure S4 Final probability distribution of sampling VQE simulation Page 12

Note S3 Impact of ansatz structure and depth on sampling performance Page 13

Figure S5 Quantum circuits for different ansatz Page 15

Figure S6 Comparison of final probability distributions for different ansatz Page 16

Figure S7 Transpiled quantum circuit for quantum hardware calculation Page 17

References Page 18

Table S1. Acronyms used in the main manuscript and its full term.

Acronym	Full Term
MTV	Multivariate
MOF	Metal–Organic Framework
THQ	Tetrahydroxy-1,4-quinone
HHTP	2,3,6,7,10,11-Hexahydrotriphenylene
COF	Covalent-Organic Framework
NP	Nondeterministic Polynomial
VQE	Variational Quantum Eigensolver
BDC	Benzene Dicarboxylate
NISQ	Noisy Intermediate-Scale Quantum
HHTT	2,3,7,8,12,13-Hexahydroxytetraazanaphthotetraphene
HHTN	2,3,8,9,14,15-Decahydronaphthalene
SPSA	Simultaneous Perturbation Stochastic Approximation
QUBO	Quadratic Unconstrained Binary Optimization

Note S1. Comparative analysis of balance cost as a function of α

To explore the potential for systematic and adaptive selection of the sensitivity parameter α in our Hamiltonian model, we performed additional classical pre-analyses. Specifically, we analyzed how the balance cost term (the only α-dependent term in our Hamiltonian model) varies across linker configurations and α values, while fixing the ratio and occupancy constraints. This assumes a situation where the correct linker ratio and unit cell sizes are satisfied, and the only variation in the Hamiltonian arises from balance cost. Instead of plotting balance cost against the Hamiltonian value, which varies with α, we plotted it against representative linker configurations to assess consistency across α values (Figure S1). This approach allows to compare how the landscape of the Hamiltonian would evolve at different α.

The result showed distinct behaviors depending on the topology. hcb-based structures, Cu-THQ-HHTP and Py-MV-DBA-COF, exhibited a V-shaped trend in balance cost at higher α values (α = 0.25 and 0.5), where the experimentally expected ground state configuration (e.g. ABABABAB) was no longer the lowest-energy state. This led to suboptimal outcomes, as the quantum circuit optimized toward a local minimum (e.g. AABBBBAA). In contrast, at lower α values (α = 0.1 and 0.01), the balance cost differences between configurations widened, increasing the distinguishability of the global minimum and enhancing VQE's ability to converge on it.

In contrast, MUF-7, which adopts an ith-d topology, exhibited an S-shaped trend in balance cost. This arises because the defined six-linker unit cell (Figure S4b) contains configurations that maintain fixed balance cost across α values due to their structural symmetry as shown in ABABAB and AAABBB in Figure R1c. The ith-d topology's edge structure consists of three topological and three spatial edges (Figure S4b) and this results in some

configurations always having zero or invariant deviations. This causes the balance cost landscape to become nearly flat at high α, increasing the risk of convergence to non-optimal states. However, at lower α values, the landscape becomes more linearly separable, improving the ability of the quantum algorithm to distinguish the ground state.

These results indicate that α plays a crucial role in shaping the Hamiltonian energy landscape and therefore directly impacts the convergence behavior of quantum optimization algorithms. We recommend conducting a pre-simulation evaluation of the balance cost landscape at various α values as a classical diagnostic tool. This strategy allows for identifying α values that preserve the correct ground state while enhancing its energy separation from suboptimal configurations. In the systems studied, $\alpha = 0.01$ consistently offered the most favorable trade-off between selectivity and convergence. While $\alpha = 0.001$ also provided similar separation, its impact on spatial adjacency terms became negligible, so $\alpha = 0.01$ was selected for subsequent simulations in this work.

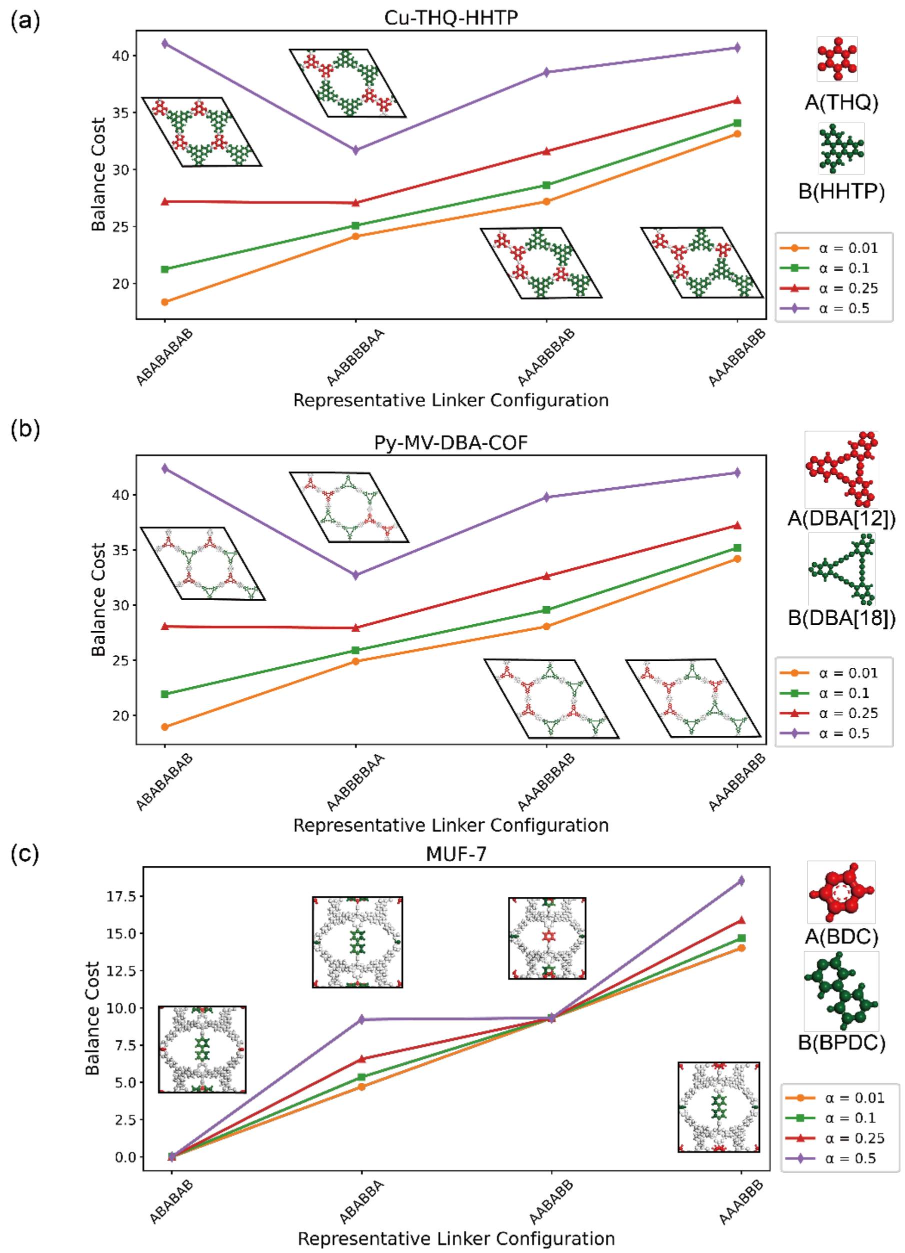

Figure S1. Balance cost trends across representative linker configurations at varying values of α in the Hamiltonian. For each system, **(a)** Cu-THQ-HHTP, **(b)** Py-MV-DBA-COF, and **(c)** MUF-7, the linker ratio and site occupancy constraints were fixed, isolating the influence of the balance cost term. Each data point represents the balance cost associated with a specific spatial arrangement of linkers, while the five curves correspond to different α values ranging from 0.01 to 0.5.

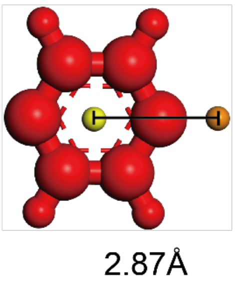

Ditopic Linkers(BDC)

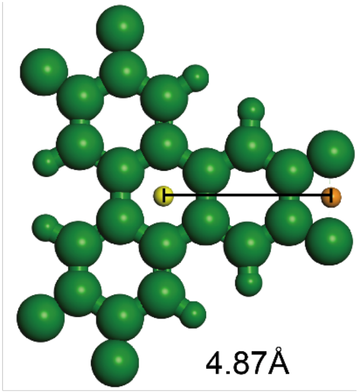

Tritopic Linkers(HHTP)

Figure S2. Illustration of the characteristic length for two types of linkers: ditopic and tritopic. The characteristic length is defined as the distance from each metal coordination site (orange) to the center of mass (yellow) of the linker. The ditopic linker BDC (red) has a characteristic length of 2.87 Å and the tritopic linker HHTP (green) has a characteristic length of 4.87 Å.

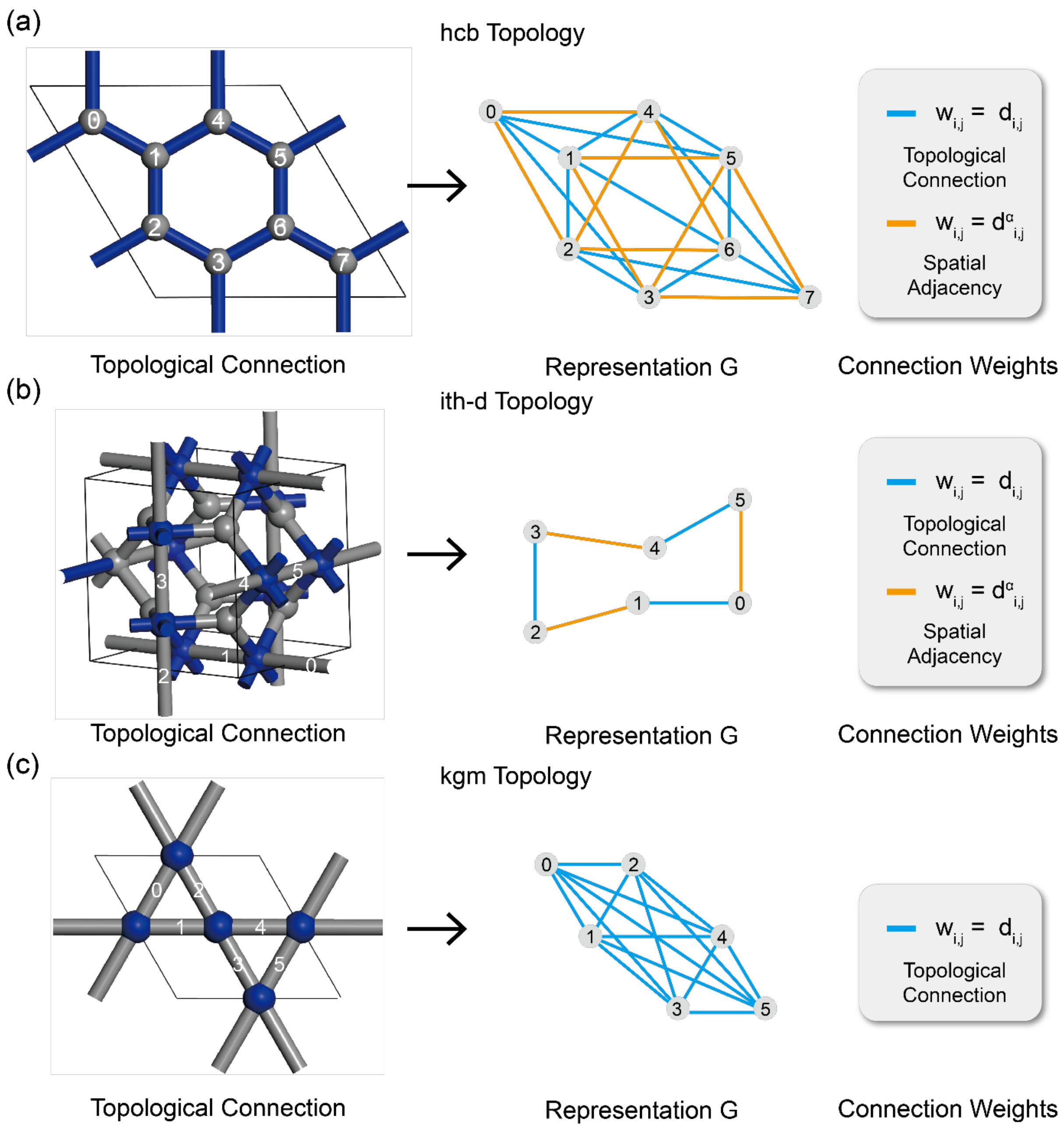

Figure S3. $G(i, j, w_{i,j})$ of candidate experimental structures for sampling VQE simulation. Framework mapping of connections, (i, j), (blue) between building block sites (grey) into a graphical representation ($G(i, j, w_{i,j})$) of (a) hcb topology (b) ith-d topology (c) kgm topology. Each connection is weighted by $w_{i,j}$, which quantifies the strength of either the direct topological connection (light blue) or the spatial adjacency (yellow).

Note S2. Computational method for Sampling VQE simulation

To simulate experimental structures, the unit cell of hcb topology was modeled as an eight-linker-site system, and kgm and ith-d topologies were assumed as six-linker-site systems. The characteristic lengths of candidate linkers, l^t, were measured by visualizing structures using Atomic Simulation Environment (ASE)[1] (Table S2). The spatial distance between nodes, $d_{i,j}$, was determined based on measured distances between nodes from the cgd format of each topology candidate obtained from the RCSR database[2] (Table S3). The sensitivity parameter, α, for each experimental structure was determined from the comparative calculations across different α values (Table S1).

Once the graph-based framework, $G(i, j, w_{i,j})$, was prepared, the Hamiltonian model was mapped into a quantum circuit using a Two-Local ansatz. The optimization process was executed on IBM Qiskit[3] using the SPSA classical optimizer with a maximum iteration count of 300. Each trial state was sampled 1024 times per iteration to approximate the expectation value of the Hamiltonian. The Minimum Eigen Optimizer was used to solve the optimization problem. The final probability distributions were obtained after 128 iterations for each structure to account for fluctuations in individual runs.

For the Sampling VQE simulation, the total Hamiltonian, $\mathrm{H(q)}$, was formulated with the addition of balancing constants for ratio and occupancy cost terms, C_{ratio} and $C_{occ.}$, as follows:

$$H(q) = C_{ratio} \sum_t \left(\sum_{i=0}^{N_i-1} {q^t}_i - n_t\right)^2 + C_{occ.} \sum_{i=0}^{N_i-1} \left(\sum_t {q^t}_i - 1\right)^2 + \sum_{G\in(i,j,w_{i,j})} w_{i.j} (L(q, G) - \bar{L})^2 \qquad (1)$$

The ratio and occupancy cost terms were assigned weighting factors of $C_{ratio} = 200$ and

$C_{occ.} = 300$, respectively, to strongly enforce these fundamental structure constraints. These weighting factors were chosen to prevent the balance cost term from dominating the total Hamiltonian, as variations in $w_{i.j}$ and $L(q, G)$ could otherwise disproportionately influence the optimization process. This ensures that the fundamental structural rules for forming a reasonable porous framework are maintained while balancing out the contributions from spatial and connectivity-based constraints.

Table S2. Characteristic lengths of candidate linkers.

MOF	Linker	l^t [Å]
Cu-THQ-HHTP	THQ	2.42
	HHTP	4.87
Py-MV-DBA-COF2	DBA[12]	8.027
	DBA[18]	10.516
MUF-7	BDC	2.869
	BPDC	5.025
SIOC-COF2	BPDA	4.6
	TPDA	6.89

Table S3. Summary of parameters for $G(i, j, w_{i,j})$

MOF	Topology	N_i	α	$d_{i,j}$ [Å]	$w_{i,j}$ [Å]
Cu-THQ-HHTP	hcb	8	1	3	3
			0.01	5.2	1.02
Py-MV-DBA-COF	hcb	8	1	3	3
			0.01	5.2	1.02
MUF-7	ith-d	6	1	3.92	3.92
			0.01	3.92	1.01
SIOC-COF2	kgm	6	1	1.5	1.5
				2.6	2.6
				3	3

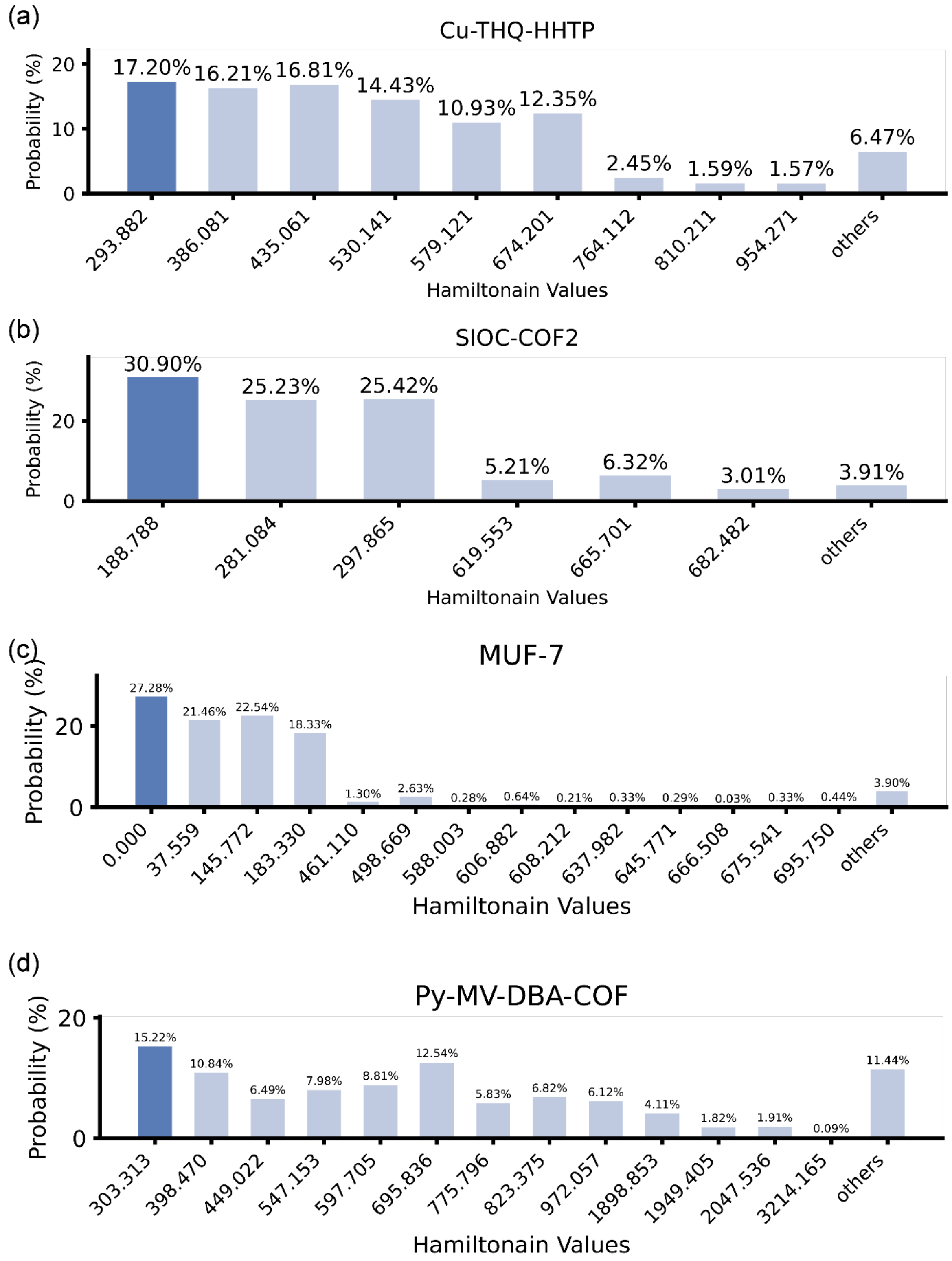

Figure S4. Final probability distribution of VQE simulation of candidate structures. The lowest Hamiltonian of each structure, marked with dark blue, corresponds to the experimental structures. For visual clarity, selective Hamiltonians are shown within individual thresholds.

Note S3. Impact of ansatz structure and depth on sampling performance

The structure and depth of the ansatz are critical factors influencing the optimization landscape and, consequently, the quality of the results in variational quantum algorithms. To examine this effect, we conducted additional VQE simulations using two more expressive ansatz configurations: 1) a three-layer Two Local ansatz, and 2) a three-layer Efficient SU2 ansatz. Among the four candidate materials evaluated in this study, we selected SIOC-COF2, which requires the fewest qubits, as a representative test case.

The single-layer Two Local ansatz consists of parameterized single-qubit Ry rotations, followed by controlled-Z (CZ) gates in a linear entanglement pattern, and a second set of Ry rotations. This circuit has 24 trainable parameters (Figure S5a). The three-layer Two Local ansatz simply stacks this configuration three times, resulting in 48 parameters (Figure S5b). In contrast, the Efficient SU2 ansatz is a widely used SU(2)-based circuit in variational algorithms[4-6], designed to efficiently explore the SU(2) Hilbert space with polynomial circuit depth. It alternates layers of single-qubit Ry and Rz rotations with entangling controlled-X (CX) gates arranged linearly. The three-layer Efficient SU2 circuit used here consists of 96 trainable parameters (Figure S5c). For each ansatz, we performed 128 independent VQE runs using the SPSA optimizer. All other parameters including iteration count and the underlying Hamiltonian model were kept consistent across experiments, as described in the Methods section.

Results are summarized in Figure S6, which presents the final probability distributions of the six lowest Hamiltonians. As the expressilbility of the ansatz increased, we observed a corresponding decrease in the average probability of measuring the lowest Hamiltonian state. Specifically, the single-layer Two Local ansatz achieved a probability of

30.9%, while the three-layer Two Local and Efficient US2 ansatz resulted in 27.41% and 4.84%, respectively. This trend reflects a trade-off, while deeper or more expressive circuits can explore a broader solution space, they also introduce a higher-dimensional parameter landscape that tend to disperse the probability distribution. As a result, the ground state configuration becomes less likely to be the dominant measurement outcome. In contrast, the shallower single-layer Two Local ansatz provides more concentrated sampling, improving the likelihood of high-probability sampling of the ground-state solution.

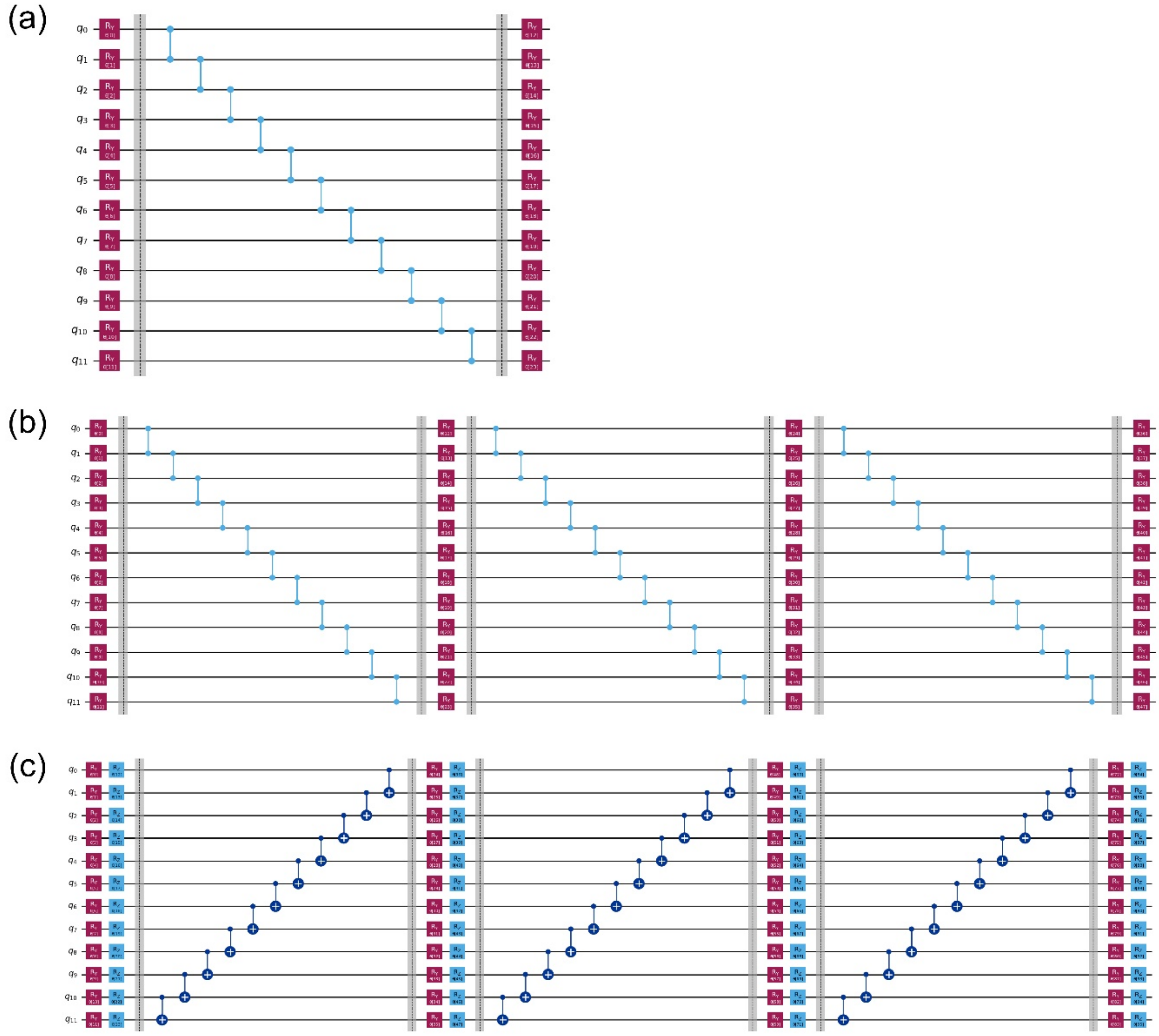

Figure S5. Quantum circuit of SIOC-COF2 within a defined unit cell consisting of six-linker site system, based on **(a)** the single-layer Two Local ansatz, **(b)** three-layer Two Local ansatz, and **(c)** Efficient SU2 ansatz.

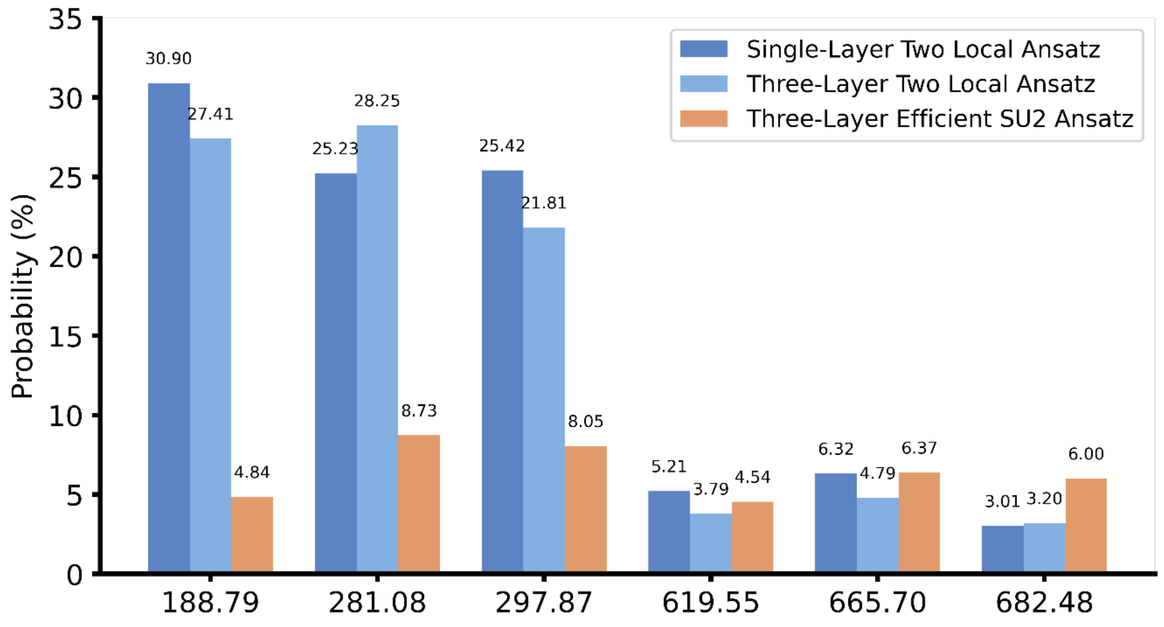

Figure S6. Comparison of final probability distributions for the six lowest-energy Hamiltonians using three different ansatz circuits applied to the SIOC-COF2 system. The distributions were obtained from Sampling VQE simulations by averaging the results of 128 independent runs for each ansatz.

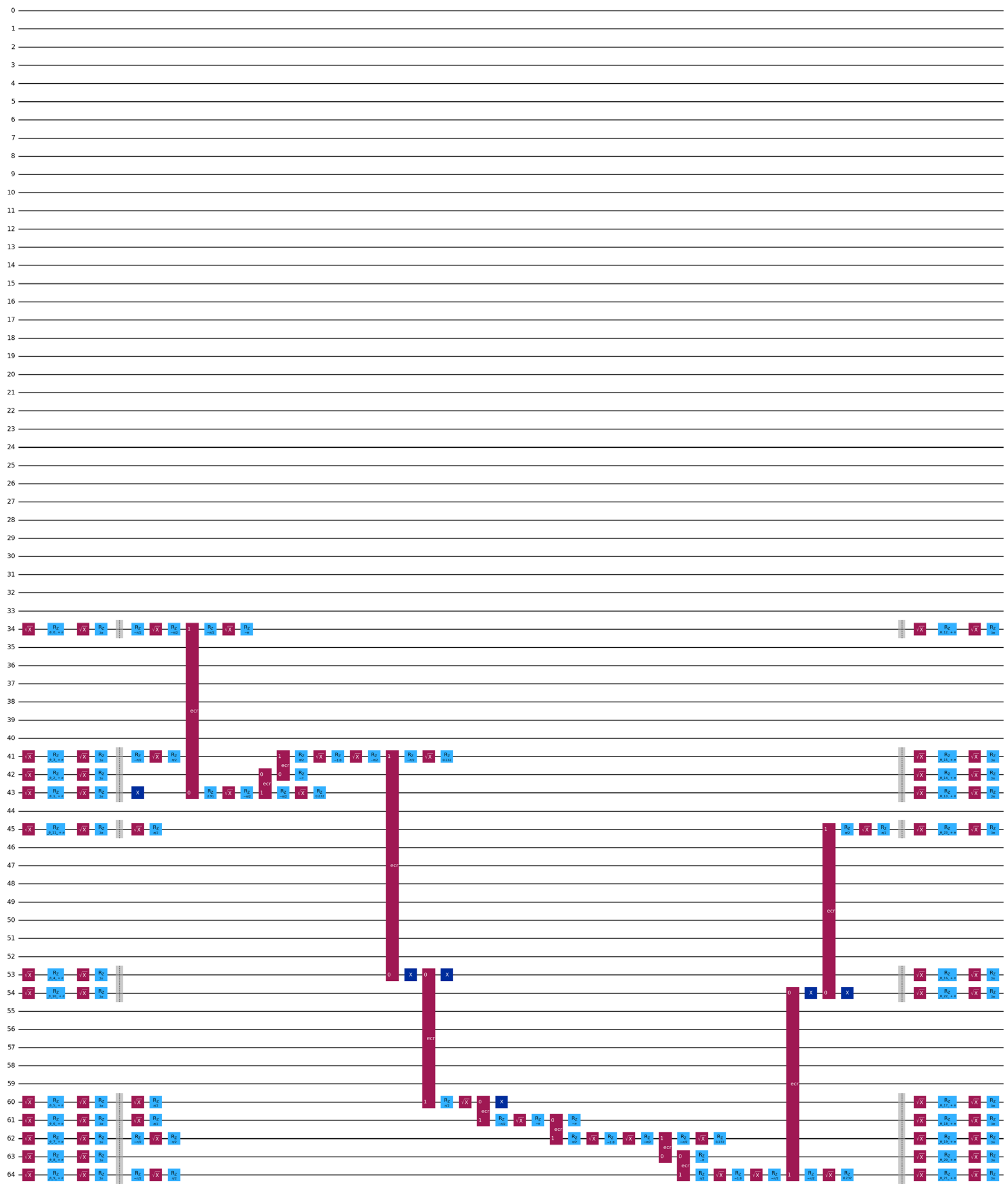

Figure S7. Input quantum circuit for the12-qubit system of SIOC-COF2. The circuit was transpiled with an optimization level of 3 and executed on ibm_kyiv.

References

(1) Hjorth Larsen, A.; Jorgen Mortensen, J.; Blomqvist, J.; Castelli, I. E.; Christensen, R.; Dulak, M.; Friis, J.; Groves, M. N.; Hammer, B.; Hargus, C.; et al. The atomic simulation environment-a Python library for working with atoms. *J Phys Condens Matter* **2017**, *29* (27), 273002. DOI: 10.1088/1361-648X/aa680e From NLM PubMed-not-MEDLINE.

(2) MICHAEL O'KEEFFE, M. A. P., STUART J. RAMSDEN, OMAR M. YAGHI. The Reticular Chemistry Structure Resource (RCSR) Database of, and Symbols for, Crystal Nets. *Acc Chem Res* **2008**, *41* (12), 1782-1789.

(3) *Qiskit: An Open-source Framework for Quantum Computing*; IBM: 2019. https://zenodo.org/records/2562111 (accessed Feb. 2025).
